# Essential Factors on Effective Response at the Onset of the COVID-19 Pandemic: Comment on "Experiences and Implications of the First Wave of the COVID-19 Emergency in Italy: A Social Science Perspective"

**DOI:** 10.34172/ijhpm.8642

**Published:** 2024-08-28

**Authors:** Jesús Cortes, Matilde Pacheco, Inês Fronteira

**Affiliations:** ^1^NOVA National School of Public Health, Public Health Research Centre, Comprehensive Health Research Centre, CHRC, NOVA University Lisbon, Lisbon, Portugal.; ^2^Instituto de Higiene e Medicina Tropical, Universidade Nova de Lisboa, Lisbon, Portugal.; ^3^NOVA National School of Public Health, Public Health Research Centre, Comprehensive Health Research Center, CHRC, REAL, CCAL, NOVA University Lisbon, Lisbon, Portugal.

**Keywords:** Italy, COVID-19, Pandemic, First Wave, Social Sciences

## Abstract

The COVID-19 pandemic in Italy is a stark reminder of the necessity of incorporating the social, economic, and political context in planning responses to public health emergencies. During the ongoing global COVID-19 crisis, it is not just crucial but a shared responsibility to supplement epidemiological approaches with insights from the social sciences. This ensures effective and equitable policies, and it is a responsibility that each of us in the field shares. This discussion is relevant and timely, relating directly to the current global crisis and its potential implications for future public health strategies. This comment underscores the key points of Masino and Enria’s paper, illuminating the importance of integrating social sciences into public health strategies, the pivotal role of inequalities in shaping pandemic experiences, and, most importantly, the profound and urgent implications for future epidemic preparedness and response. The urgency of these implications cannot be overstated, and we must act on them swiftly and decisively.

## Background

 During the COVID-19 pandemic, countries strived to achieve an efficient and organized response between the governmental and social parties. For example, China, Korea, and Singapore effectively controlled their outbreaks with strict containment measures and efficiently managed infected individuals.^1^ In the other hand, the United States, the United Kingdom, and France, which used mitigation policies, experienced less success, with the epidemic persisting or worsening.^1^

 In discussing Italy’s experiences during the pandemic’s initial wave, Masino and Enria^2^ have presented unique societal stances. By examining the intersection of social, economic, and political dimensions, the authors provide a nuanced understanding of how these factors influenced the Italian response and the outcomes, offering a fresh and intriguing perspective.

 On February 20, 2020, a 38-year-old man from Codogno, Italy, was officially the first positive case of COVID-19 in the country. This marked the beginning of a rapidly escalating health crisis that would soon overwhelm the country’s healthcare system.

 The transmission in Northern Italy had been underway, unnoticed, since at least the beginning of January 2020 or possibly Fall 2019. Evidence of the sustained spread of the disease only emerged in February, prompting emergency lockdown measures. By March 16, 2020, Italy had 27 980 recorded cases and was second only to China in cumulative mortality. A year later, Italy had the seventh-highest total cases and the third-highest deaths per 100 000 population worldwide. The severity of Italy’s experience with COVID-19, ranking seventh-highest in total cases and third-highest in deaths per 100 000 population worldwide, is not just crucial, but a stark testament to the gravity of the pandemic’s impact on Europe.^3^

 Masino and Enria^2^ emphasize that while Italy had a relatively well-resourced healthcare infrastructure, the pandemic exposed deep socioeconomic inequalities and governance issues that exacerbated the crisis. Similarly, a study by Giannoni et al showed that access to healthcare declined, particularly in regions with lower income and higher inequality, worsening disparities for vulnerable groups such as the elderly and migrants.^4^

## Relevance of Social, Political, and Economic Factors

 The COVID-19 pandemic has highlighted the importance of social, political, and economic contexts, including the impact of policy decisions, the relationship between marginalization and infection risk, and the historical roots of mistrust in response measures like vaccination.^5^

 The authors employ a qualitative methodology, drawing on 29 testimonies gathered through semi-structured interviews and written accounts. This approach is well-suited to capture the lived experiences of individuals across diverse social groups, regions, and employment sectors. By focusing on personal testimonies, the authors reveal the ways in which the pandemic has affected everyday life, from the pressure on healthcare workers to the economic difficulties faced by those who work informally. By including varied perspectives, the authors ensure that their analysis reflects the heterogeneous nature of Italian society. This methodological approach enhances the reliability and depth of their findings, offering rich, contextualized insights into the impact of pandemic and identify recommendations for future interventions.

 The rapid COVID-19 spread in early 2020 overwhelmed Italy’s healthcare system, particularly in the initially affected North.^6^ The subsequent lockdown triggered a mass migration to the South, potentially spreading the virus highlighted the urgent need for a robust public health response. The pandemic severely impacted the economy, exacerbated inequalities, and complicated care for vulnerable populations like the elderly. This situation was mirrored across Europe, where older adults experienced decreased social contacts, increased loneliness, and deteriorated mental health.^7^ Financial difficulties arose from pandemic-related expenses despite stable pension incomes.^7^ Reduced physical activity and insufficient e-healthcare also affected older people, highlighting the need for better public health responses and support systems in the future.^7^

## Regional Disparities and Healthcare Shortage

 The regional disparities within Italy are another significant finding of the study. The north-south divide in healthcare infrastructure and resources has been clear during the pandemic. Although northern regions, such as Lombardia, were initially overwhelmed by the large number of cases, they benefited from better-equipped health facilities and a more responsive health administration. In contrast, southern regions struggled with limited intensive care units capacity and a slower governmental response, which exacerbated the crisis and led to higher mortality rates and more severe socioeconomic impacts. The swift outbreak of COVID-19 in Italy at the beginning of 2020 caught the healthcare system off guard, leading to a shortage of beds, staff, and personal protective equipment (PPE). The authors highlight that these shortages were more pronounced in southern regions. Family doctors, who play a critical role as primary healthcare providers, have faced significant delays in receiving PPE, mainly because they are self-employed and not directly integrated into the public healthcare system. This lack of protection not only increased the risk of virus transmission in communities but also placed healthcare workers in dangerous conditions, undermining the overall effectiveness of the healthcare response.

 The lockdown of northern regions led to a mass exodus of people to the south, potentially spreading the virus further. The suspension of non-essential medical procedures and the fear of infection disrupted care for patients with other diseases.

## Economic and Social Challenges

 The economic impact of the pandemic was severe, with a contraction in gross domestic product and widespread job losses, particularly in the tourism and entertainment sectors. The self-employed and those in informal work were particularly vulnerable, with a delay in government support measures leaving many without income. There were concerns about the ability of people living in overcrowded conditions to follow social distancing measure and the care of the elderly, who were at high risk from COVID-19, was complicated by the need to balance their protection with the prevention of isolation and the disruption of intergenerational support networks.

## Institutional Trust

 The institutional trust is another crucial aspect that the paper addresses. Historically, trust in Italian institutions has been relatively low, and this distrust has been a significant obstacle during the pandemic. Masino and Enria point to inconsistent and often contradictory communication from authorities, which has further eroded public trust. For example, the premature abandonment of stay-at-home orders and inconsistent implementation of testing protocols have created a climate of uncertainty and fear. This lack of clear and reliable communication from government and health authorities has undermined public adherence to health directives, complicating efforts to control the spread of the virus. Zhai et al study suggests that trust generated by successful government anti-epidemic measures is enduring, emphasizing the need to bolster trust among socially vulnerable groups.^8^

## Key Findings

- The rapid onset of the COVID-19 pandemic in Italy followed a delay in recognizing the spread of the virus in January 2020, allowing unchecked exponential growth, particularly in Lombardia.
- While Lombardia and the North have well-resourced health systems, the same cannot be said for the rest of Italy, where the healthcare system often reflects decades of divided growth.
- The publicized announcement of a lockdown in Lombardia led thousands of residents from North to the South of Italy, potentially spreading the virus further.
- The population of southern Italy, with its dependence on informal work, was particularly vulnerable.
- Institutional actions were perceived as favoring the North, intensifying feelings of injustice and increasing the risk of organized crime taking advantage of struggling businesses in the South.
- Healthcare workers were particularly affected, facing PPE shortages and being required to work despite potential exposure to the virus.
- Low institutional trust and contradictory communication from the government resulted in reduced adherence to public health guidelines.

## What Was Learned?

 This article is a call for a rethinking of traditional public health strategies to include broader societal contexts, ensuring that future responses to health crises are more equitable and effective.

 What happened in Italy allows us to reflect on the need for a new public health that integrates not only health services and responses but also the entire ecosystem and environment surrounding the human experience.^9^ This postulate agrees with the World Health Organization’s (WHO’s) guidelines, which call for one health to be considered one.^10^ The discussion surrounding COVID-19 has propelled conversations about the One Health approach, emphasizing the interconnectedness of human, animal, and environmental health.^11^ This holistic perspective acknowledges the complex web of factors influencing public health outcomes and underscores the importance of interdisciplinary collaboration and global cooperation.^12^ In light of these developments, it becomes evident that the need to rethink public health responses extends beyond individual countries.^13^ By embracing a holistic approach and by fostering collaboration at both global and regional levels, we can better address the challenges posed by pandemics like COVID-19 and promote health and well-being on a global scale.^12^

 Masino and Enria^2^ argue that public health strategies often focus narrowly on epidemiological data, such as infection rates and hospital capacity, without sufficiently considering the broader social determinants of health. These socioeconomic determinants of health created a situation where certain populations were disproportionately affected by the pandemic, both in terms of health outcomes and socioeconomic consequences, this is also referred in the study conducted by Kapiriri and Ross.^14^

 A systematic review by Fronteira et al highlights the profound impact of Public Health Emergencies of International Concern on healthcare workers’ mental and physical health.^15^ The consequences include increased absenteeism, service disruption, and turnover, challenging the resilience of health systems.^15^ Prioritizing healthcare worker well-being and establishing robust support systems are essential during emergencies.^15^ Testing strategies should consider the need to detect asymptomatic cases to control spread. Socioeconomic factors, such as living conditions and employment status, should be considered when assessing vulnerability to infection. In regions with significant inequalities, context-specific measures are essential to increase the efficacy and effectiveness of interventions. For instance, providing targeted financial support to informal workers or ensuring access to healthcare for undocumented migrants can help mitigate the impact of future pandemics. Caring for the elderly requires a nuanced approach to balancing protection with psychosocial needs.

 The experiences of Italy during the first wave of COVID-19 serve as a powerful reminder of the critical role of trust and communication in public health responses. Clear and consistent communication is essential for building trust and ensuring adherence to public health measures which should prioritize not only individual protection but also the well-being of the broader community.^16^ Effective communication strategies should be tailored to consider not only information about virus transmission but also psychosocial aspects, such as trust and moral values. Furthermore, involving diverse stakeholders, including community representatives and social scientists, is essential to enhance adherence to public health measures. Plagiaro et al argue that by incorporating these insights into interventions, behavioural changes required to control the COVID-19 outbreak and safeguard the well-being of individuals and communities worldwide can be effectively induced.^16^

## Conclusion

 In sum, the COVID-19 pandemic in Italy demonstrates that public health emergency responses must consider social, economic, and political contexts. Epidemiological approaches with traditional risk profiles, which predominantly focus on age and pre-existing health conditions alone are insufficient; social science insights are necessary for effective and equitable policies. The paper provides recommendations for policy-makers, public health officials and researchers, emphasizing clear communication, healthcare workers protection, and socioeconomic vulnerability assessment. It also underscores the need for further research, including comparative studies of successful local governance of epidemics.

## Ethical issues

 Not applicable.

## Competing interests

 Authors declare that they have no competing interests.
